# Supplementary material for: Content-rich biological network constructed by mining PubMed abstracts
Source: BMC Bioinformatics. 2004 Oct 8;5:147. doi: 10.1186/1471-2105-5-147 (PMC528731; doi:10.1186/1471-2105-5-147)
Supplement: Additional File 2 — The original results of the above study (non-essential files are deleted to keep the file size under the limit set by BMC bioinformatics). [file 1471-2105-5-147-S2.bz2 › chilibotAdditionalFile2/dip10/253ID7809159E1011/html/CKS1_CDK2.html]

 


 **CKS1** and **CDK2** 
  
Found 11 abstracts in PubMed, retrieved 10.  
 

 What does Google say? 
 PDF only 
| .edu only 

---

**Interactive relationship** (e.g. stimulation, inhibition, etc)

**Neutral relationship**- The interaction of Skp2 with the substrate is further strengthened by the association of the Cdk binding site of  **Cks1**  with  **Cdk2**  cyclin E, to which phosphorylated p27 is bound.  Ref: 12140288 J Biol Chem, 2002
- **CDK2**  binds the Cks  [ **CKS1** ]  single domain conformation and interacts with conserved hydrophobic residues plus His 60 and Glu 63 in their closed beta hinge motif conformation.  Ref: 8601310 Cell, 1996
- One face of the complex exposes the sequence conserved phosphate binding region on Cks  [ **CKS1** ]  and the ATP binding site on  **CDK2** , suggesting that CKs  [ **CKS1** ]  may target  **CDK2**  to other phosphoproteins during the cell cycle.  Ref: 8601310 Cell, 1996
- iii in vitro, the phosphorylated forms of CDK1 and  **CDK2**  were preferentially linked to CKS  [ **CKS1** ] .  Ref: 10360827 Int J Cancer, 1999
- The beta hinge opening to form the Cks  [ **CKS1** ]  beta interchanged dimer sterically precludes  **CDK2**  binding, providing a possible mechanism regulating  **CDK2**  Cks  [ **CKS1** ]  interactions.  Ref: 8601310 Cell, 1996
- The 2.6 Angstrom crystal structure for human cyclin dependent kinase 2  [ **CDK2** ]   **CDK2**  in complex with CksHs1, a human homolog of essential yeast cell cycle regulatory proteins suc1 and  **Cks1** , reveals that CksHs1 binds via all four beta strands to the kinase C terminal lobe.  Ref: 8601310 Cell, 1996

**Non-interactive relationship** (e.g. studied together, co-existance, homology, etc.)

- On the basis of the structure of  **cdk2**  CksHs1 complex and on our kinetic results, we propose that the binding of Cks  [ **CKS1** ]  proteins to C lobe of  **cdk2**  is stabilized by the presence of cyclin A.  Ref: 9760264 Biochemistry, 1998
- Despite the high degree of homology between the nine known CDKs, only CDK1,  **CDK2**  and, to a lesser extent, CDK3 are able to bind to the suc1 Cks  [ **CKS1** ]  proteins.  Ref: 12007796 Biochim Biophys Acta, 2002
- The ubiquitylation of p21 in vitro required the supplementation of all components of the SCF complex, as well as of  **Cks1**  and  **Cdk2**  cyclin E.  Ref: 12730199 J Biol Chem< MedlineTA>J Biol Chem, 2002
